# Supplementary figures and images for: The importance of oxytocin neurons in the supraoptic nucleus for breastfeeding in mice
Source: PLoS One. 2023 Mar 17;18(3):e0283152. doi: 10.1371/journal.pone.0283152 (PMC10022762; doi:10.1371/journal.pone.0283152)

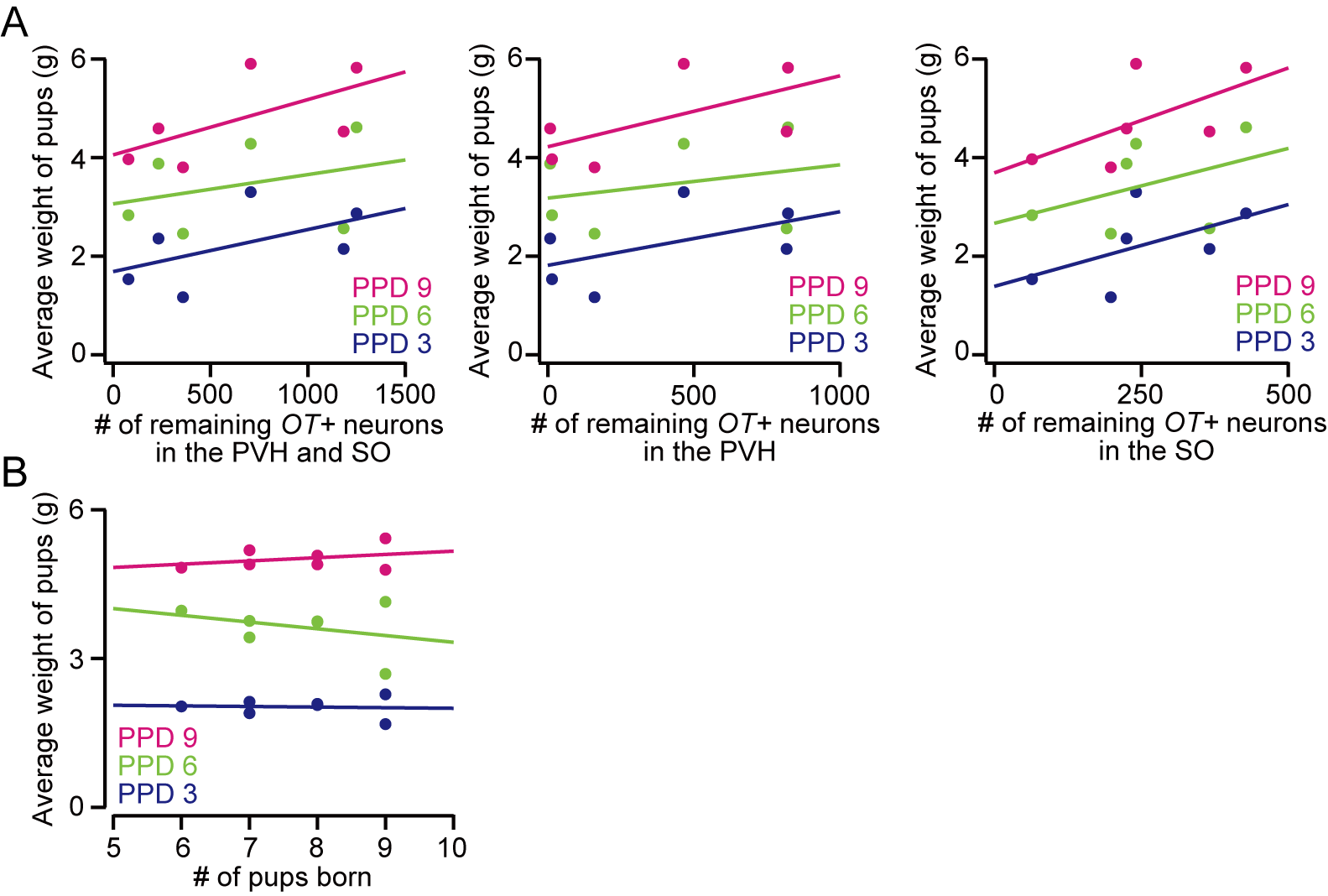

Supplement: S1 Fig — (A) Relationship between the average weight of pups and the number of remaining OT+ neurons in the PVH and SO (left), PVH (middle), and SO (right). Data was obtained from six OTflox/− mice that received AAV-Cre injection into the bilateral PVH and SO. Left, R2 = 0.28, 0.10, and 0.38 for PPD 3, PPD 6, and PPD 9, respectively, p > 0.19 for all PPDs. Middle, R2 = 0.27, 0.07, and 0.37 for PPD 3, PPD 6, and PPD 9, respectively, p > 0.20 for all PPDs. Right, R2 = 0.29, 0.18, and 0.37 for PPD 3, PPD 6, and PPD 9, respectively, p > 0.19 for all PPDs. (B) In our datasets, the number of pups born was not strongly correlated with the development of pups (R2 = 0.01, 0.10, and 0.10 for PPD 3, PPD 6, and PPD 9, respectively. p > 0.47 for all PPDs). Data were obtained from seven OTflox/− mice that received vehicle injection into the bilateral PVH and SO. Note that data points from two mothers of 8 litters nearly overlap. (TIF) [file pone.0283152.s001.tif]
