# Supplementary material for: The importance of oxytocin neurons in the supraoptic nucleus for breastfeeding in mice
Source: PLoS One. 2023 Mar 17;18(3):e0283152. doi: 10.1371/journal.pone.0283152 (PMC10022762; doi:10.1371/journal.pone.0283152)
Supplement: Attachment — Submitted filename: Hagihara et al response to the reviewers.docx [file pone.0283152.s002.docx]

We greatly thank the editor and the reviewers for their enthusiasm about our study, and the constructive suggestions. Here we have conducted two new experiments and two data analyses that, we believe, will satisfactorily address the reviewer’s comments and substantially improve our manuscript in terms of biological significance and clarity of the text. Here, we summarize the major points and provide point-by-point responses to the reviewer’s comments.

**Additional experiments performed:**

1) PVH-specific *OT* cKO to test the importance of OT secretion from PVH in pregnancy, delivery, and milk ejection (Response #1-1, #2-1, #2-10), as reported in **new Fig 6**.
2) Maternal behavior assay with the virgin females of *OT* cKO in PVH and SO (Response #2-2), as reported in **new Fig 3**.

**Additional data analyses performed:**

1) Analyzing whether the number of remaining *OT+* neurons in the PVH and SO predicts pup growth (Response #2-12), as reported in **new S1 Fig A**.
2) Analyzing the correlation between the litter size and the pup growth (Response #2-14), as reported in **new S1 Fig B**.

**Point-by-point responses**

Reviewer #1: The paper by Hagihara et al. reports interesting new findings on the role of oxytocin in regulating parturition, breastfeeding and maternal behavior in female mice. The Authors are leaders in this field of research and have published outstanding papers on oxytocin and reproduction. Here, by selectively deleting oxytocin, or oxytocinergic neurons, in hypothalamic PVN and/or SO nuclei, they confirm that oxytocin is dispensable for parturition and maternal care but also show that the milk ejection role of oxytocin is specifically supported by SO oxytocinergic neurons. Collectively, the paper is well conceived and well written, the methods are adequate. However, some points should be tackled by the Authors before recommending publication.
Major point
In my opinion, the major result of the paper, that oxytocin neurons of the hypothalamic SO nucleus are specifically involved in milk ejection, especially at the onset of lactation, should be confirmed by selectively deleting oxytocin in the PVN. In these mice, according to the Authors’ hypothesis, a normal, or almost-normal, breastfeeding behavior should be observed. This is the most outstanding result of the paper, and the title could be changed or implemented

Response #1-1

We are greatly thankful to this reviewer for a very positive evaluation of our main aims and conclusions. As suggested, we performed PVH-specific cKO of the *OT* gene and examined the influence on breastfeeding (**Additional Experiment #1**). As suggested, we did not find major defects in the PVH-specific cKO mothers. We summarized our findings in the **new Fig 6**. Also, we modified the title to emphasize the roles of OT neurons in the SO.

Minor points
Throughout the manuscript the Authors claim to possible compensatory mechanisms (“genetic compensation”, “enhance the expression of related genes” … which genes?), that in fully oxytocin KO mice could hinder some phenotypes. They should be clearer, and specifically explain what they mean.

Response #1-2

We greatly thank the reviewer for this important comment. Decades of studies of reverse genetics in flies, mice, and other model animals have suggested the ability of an organism to maintain fitness in the presence of harmful mutations, termed genetic robustness. Although this can be achieved by multiple strategies, recent seminal studies emphasized the significance of transcriptional adaptation, termed genetic compensation, in which a mutation can lead to the increased expression of related genes that are themselves able to assume the function of the mutated gene (e.g., El-Brolosy et al., *Nature* 568: 193, 2019). In the context of whole-body *OT* KO mice, the lack of robust deficits in maternal behaviors was hypothesized to be due to compensatory mechanisms through activation of the Oxtr by vasopressin or via some compensation mechanisms during development (Winslow JT & Insel TR, *Neuropeptides* 36: 221, 2002). We recently showed that *OT* cKO mice exhibited elevated food intake, which was not observed in whole-body *OT* KO mice. This phenotypic difference might be explained by genetic compensation in the whole-body *OT* KO mice (Inada et al., *eLife* 11: e75718, 2022), although we have not identified the exact gene(s) that show enhanced expression to suppress the overeating in the *OT* KO mice. In the revised manuscript, we textually clarified these points (lines 366–375).

In this context (Introduction), why “PVH-specific OT cKO mice show hyperphagic obesity” is not clear to the reader if the Authors do not specify that OT also has a role in food intake and energy balance in adult animals.

Response #1-3

As suggested, in the revised manuscript, we explained the involvement of OT in the homeostasis of body weight (lines 54–57).

The wide readership of the Journal may be not so confident with the mouse models created and studied in the present investigation. In addition to this, the Materials and Method section is at the end of the manuscript. So, a few sentences in the Result section, better describing the procedures used for obtaining the selective conditional KO mice, could render the paper easier to read and meaningful.

Response #1-4

We thank this important comment. In the revised manuscript, we explained more carefully how we achieved *OT* cKO in the Result, in addition to the Materials and Methods (lines 77–84 and 430–434).

When discussing the possible effects of oxytocin on dopaminergic neurons of the ventral tegmental area and rewarding mechanisms, but also concerning data obtained in the present paper, the Authors should also take into consideration the possibility that some phenotypes could be due to a some extent to cutting of afferent, or efferent, projections to, or from, the SO and/or PVN nuclei and to the involvement of glial cells.

Response #1-5

Although we do not fully exclude the possibility of cutting some afferent or efferent connections of OT neurons during our viral injections, our negative control experiments performing the same surgical procedures but just injecting saline (vehicle) showed no phenotype. Nevertheless, the viral transduction could selectively recruit activation of glial cells, which may underly some of the observed phenotypes. We carefully discussed these points in the revised manuscript (lines 404–408).

Reviewer #2: Oxytocin is necessary for milk let-down and facilitates maternal retrieval in rodents in the transition from nulliparous to parental. The main sources of oxytocin are the paraventricular nucleus of the hypothalamus and the supraoptic nucleus of the hypothalamus. In the mid-1990’s the first knockout studies of oxytocin demonstrated an absence of milk-letdown and impairments in social behavior in congenital whole-body knockouts. In the current report, the authors evaluate the site-specific role of oxytocin using conditional genetic strategies in primiparous female mice.
Strengths:
This is an interesting report that contributes to our understanding of different hypothalamic populations in physiology and behavior.
Major weaknesses:
On line 139-140, the authors state: “ These results suggest that OT+ neurons in the PVH and SO show differential contributions to pup survival.” They do not have a full dataset to support this claim. They do show that conditional deletion of OT from the SO is sufficient to recapitulate deletion from whole body (prior literature) or their conditional deletion from SO AND PVH. There is a missing experiment and result: to support their claim as quoted above, they need to show that conditional deletion of OT from the PVH does not recapitulate the phenotype. This is a likely outcome, but not demonstrated conclusively here.

Response #2-1

We greatly thank the reviewer for a very positive evaluation of our main aims and constructive suggestions. We conducted the PVH-specific *OT* cKO experiment to examine the relevance of *OT* neurons in the PVH (**Additional Experiment #1**). As shown in the **new Fig 6**, we did not find significant defects in the PVH-specific cKO.

Additionally, the by using primiparous females during retrieval testing and not virgins, the manuscript fails to capture the impact of oxytocin in the transition from inexperienced to experienced maternal retrieval behavior. It is well-established that inexperienced virgins are likely to ignore pups in their first interactions with them. However, once pregnant and delivered, primiparous dams very quickly learn to retrieve pups. Elevated estrogens from pregnancy and enhanced oxytocin seem to facilitate this process. Once maternal, retrieval behavior is established and less vulnerable to oxytocin manipulations. In this report, the authors have chosen to test their hypothesis in females who have gone through pregnancy- females who will already behave maternally and for which oxytocin is less important. Had they really wanted to evaluate a site specific contribution of the SON or PVN OT to pup retrieval, they should have tested their retrieval behavior in virgins. Contextualizing their findings with others’ work in this area should include a discussion of Rich et al (Rich ME, deCárdenas EJ, Lee HJ, Caldwell HK (2014) Impairments in the Initiation of Maternal Behavior in Oxytocin Receptor Knockout Mice. PLOS ONE 9(6): e98839, and Carcea et al (Carcea, I., Caraballo, N.L., Marlin, B.J. et al. Oxytocin neurons enable social transmission of maternal behaviour. Nature 596, 553–557 (2021). <https://doi.org/10.1038/s41586-021-03814-7>)

Response #2-2

We deeply thank these constructive suggestions. During the revision, we re-generated PVH and SO *OT* cKO virgin females and examined their performance of the alloparental behaviors after the maternal learning paradigm described in Carcea et al., 2021 (**Additional Experiment #2**). We found that those cKO virgin females ignored pups at a higher ratio compared to the control virgins. We summarized our findings in the **new Fig. 3**. We also appropriately cited the relevant papers to contextualize our data.

Minor weaknesses:
In the abstract, the language ending on line 38 should be clarified: “We found that cell ablation of oxytocin neurons leads to no additional abnormalities over the oxytocin conditional knockout, suggesting that non-oxytocin ligands expressed by oxytocin neurons have negligible functions” on the responses measured in this report. “on the responses measured in this report” should be added as clarifying language.

Response #2-3

According to the suggestion, we modified the text in the revised manuscript (line 37).

The presentation of the figures makes it unclear if the animals used in figures 1 and 2 are the same as in figures 4 and 5. If they are the same, it makes more sense to combine the figures. If they are not the same, then it would be important to show validation of cKO for this additional cohort of animals. If the figures get unwieldy with multiple panels, the following current figure panels seem unnecessary: Figure 1K, Figure 2I, Figure 3H, Figure 4C, Figure 5C

Response #2-4

We greatly appreciate this suggestion. All mice used in Figures 4 and 5 were the same as in Figures 1 and 2, respectively. Therefore, the efficiency of *OT* cKO in Figures 4 and 5 was validated. We apologize that our original Figure orders are misleading. In the revised manuscript, we reorganized the figure order such that the data originating from the same cohort are presented sequentially in one or two adjacent figures. In addition, we clearly described the animals used in each Figure legend.

More details of methods should be included instead of solely referencing prior papers. For example, for the ISH, probe details should be included.

Response #2-5

According to the suggestion, we improved the Materials and Methods section in the revised manuscript.

It is unclear why the OTflox/- was necessary or an improvement over OTflox/flox. The rationale for this choice should be explained.

Response #2-6

We chose the *OT* *flox/null* model to increase the efficiency of Cre-mediated KO. If we had used *OT* *flox/flox* mice, which have very high *OT* gene expression levels, a small fraction of the *flox* alleles that do not experience recombination would easily mask the actual phenotypes. Many influential papers have utilized *flox/null* models as conditional knockout (for example, Kwan et al., *Genesis* 39: 10**,** 2004; Pelosi et al., *PLoS ONE* 10: e0136422, 2015). In the revised manuscript, we added careful explanations in the Materials and Methods to clarify these points (lines 430–434).

How were cells counted? How many sections per side per animal?

Response #2-7

As described in the Materials and Methods section, 20 μm coronal sections containing both hemispheres of PVH and SO were obtained. The number of sections per animal was typically 32, covering 640 μm along the anterior-posterior axis. After the *in situ* hybridization, *OT+* neurons were counted manually using the ImageJ Cell Counter plugin by an experimenter who was blind to the experimental conditions. We improved the Materials and Methods section in the revised manuscript to explain the procedure more clearly (lines 515–517 and 523–525).

“In situ hybridization” would be a better section heading in the methods than “histochemistry”

Response #2-8

We modified the text as suggested in the revised manuscript.

The statistical analysis for pup growth should be a repeated measures ANOVA assuming that the animals are traceable as individuals overtime.

Response #2-9

We thank this important suggestion. As suggested, we used two-way ANOVA with repeated measurements to test whether development of pups of +Cre mothers was significantly different from that of vehicle mothers. We also clearly described which statistical test was used in each figure legend.

The cKO model uses oxytocin floxed in the context of an Oxt knockout a allele bred with males who are wild type. This breeding strategy would produce wild-type and heterozygous offspring. It is unclear what the impact of an interaction between pup genotype and mom genotype might be. This should be addressed in the discussion.

Response #2-10

We thank this important comment. Given that we crossed *OT* *flox/null* females with wild-type males, the genotype of the pups should be either *flox/+ or +/null*. In the PVH-specific *OT* cKO (**Additional Experiment #1**), we analyzed the genotype of pups. We found that both genotypes appeared at a similar ratio in both controls and +Cre mothers, suggesting that the genotypes of pups had negligible influence on our results. Regarding maternal behaviors, we all used wild-type foster pups for the assay. We described these points in the revised Materials and Methods (lines 469–475).

In figure 2 the authors use oxytocin in situ hybridization to evaluate the effectiveness of the caspase virus which clearly shows a reduction in oxytocin. However, their claim is that the caspase method reduces the oxytocin cell population rather than just the oxytocin mRNA. Ideally, they would show evidence of cell loss rather than just oxytocin mRNA loss. Is there a significant reduction in the DAPI positive cell density in the PVN and SON?

Response #2-11

We appreciate this important comment. Induction of apoptosis by caspase-3 has been intensely studied and well-described, and according to the original paper of *AAV-flex-taCasp3-TEVp* (Young et al., *Cell* 153: 896*,* 2013), injection of the AAV into the HEK293T cells expressing Cre-GFP evoked Cre-dependent cell death as expected. We estimate that the reduction of DAPI+ cells in the PVH and SO in our experimental conditions is too small to be quantitatively addressed because i) the RNA sequencing data showed that *OT+* neurons merely occupy ~3% of the PVH neurons (Xu et al., *Science* 370: 313, 2020); ii) DAPI also labels non-neuronal cells; and iii) quarter to half of the OT neurons remained. We carefully discussed these points in our revised manuscript (lines 233–236).

There appears to be a lot of variability in the pup growth across ages in virus treated females. Given that the remaining OT+ neurons are associated with pup survival, it would be interesting to probe if the remaining oxytocin neurons in the PVN or SON of dams predict pup growth for pups that do survive.

Response #2-12

According to the suggestion, we conducted an additional data analysis, aiming to show the relationship between the pup growth and the number of remaining *OT+* neurons (**Additional Data Analyses #1**). As summarized in S1 Fig. A, the number of remaining *OT+* neurons modestly correlated with the average weight of pups. We described the finding in the Results section (lines 138–139).

The lack of a virus control is unfortunate.

Response #2-13

We understand that this is a comment on a potentially harmful effect just by injecting AAV. Regarding the *AAV-flex-taCasp3-TEVp* injected into *OT-Cre* mice, in a different biological context, we observed a selective reduction of *OT* mRNA without affecting the surrounding *vasopressin* mRNA expression (data not shown), supporting that the virus alone (without Cre-based recombination) does not affect the neurons in the injection site. Regarding the *AAV-Cre*, we recently showed that injection of this virus into the PVH of wild-type mice exhibited no phenotype, while the injection into the *OT flox/null* mice resulted in hyperphagic obesity (Inada et al., *eLife* 11: e75718, 2022), supporting that the virus alone has no harmful effect in the injection site. We included these points in the revised manuscript (lines 404–408).

Were the litters normalized to the same size? Litter size impacts pup growth- too few pups and too many pups reduce per pup weight. This should probably be modeled. This is particularly important due to the influence of ventral stimulation (intensity of which is influence by the number of pups) which stimulates the Ferguson reflex for milk let-down. Is it possible that the KO is less able to modulate pup growth based on litter size?

Response #2-14

We appreciate this important suggestion. We did not normalize the litter size. As suggested, we conducted an additional data analysis to show the relationship between the litter size and the pup growth (**Additional Data Analyses #2**). As summarized in S1 Fig. B, in our dataset, the number of pups born was not correlated with the average weight of pups. We described this result in the Materials and Methods (lines 464–466).
