# Supplementary material for: The importance of oxytocin neurons in the supraoptic nucleus for breastfeeding in mice
Source: PLoS One. 2023 Mar 17;18(3):e0283152. doi: 10.1371/journal.pone.0283152 (PMC10022762; doi:10.1371/journal.pone.0283152)
Supplement: Attachment — Submitted filename: Hagihara et al response to the reviewers.docx [file pone.0283152.s003.docx]

We express our sincere gratitude to the editor and the reviewers for their favorable assessments of our revised manuscript and for providing constructive feedback. In response to the Reviewer #2's comment, we have replaced the statistical methodology used for multiple figures from the *t*-test to a non-parametric U-test. This modification will enhance the statistical accuracy of our manuscript.

**Additional data analyses performed:**

1) Re-evaluate the significance of our finding by a two-sided Mann–Whitney U-test.

**Point-by-point responses**

Reviewer #2: The authors have adequately addressed the original reviews. In the new Figure 3 F, the distribution of the data indicate the possibility that the data needs a non-parametric mann-whitney U test rather than a parametric t-test.

Response:

We are greatly thankful to this reviewer for a positive evaluation of our revision and for providing invaluable suggestions on the statistical analysis. Following a comprehensive analysis of the data structure, we have made the decision to replace the student t-test with a two-sided Mann-Whitney U-test for the following data sets: Fig. 1D, E, F, Fig. 2D, F, Fig. 3C, E, F, G, Fig. 4D, E, Fig. 5D, F, Fig. 6C, D, Fig. 7C, D. It is our belief that this alteration will lead to an enhancement in the statistical accuracy of our results. Importantly, our original conclusions remain unchanged.
